# A Ruptured Digital Epidermal Inclusion Cyst: A Sinister Presentation

**DOI:** 10.1155/2016/9035246

**Published:** 2016-06-22

**Authors:** Iain Bohler, Phillip Fletcher, Amanda Ragg, Andrew Vane

**Affiliations:** Orthopaedic Department, Tauranga Hospital, Cameron Road, Tauranga, Bay of Plenty 3112, New Zealand

## Abstract

Epidermal inclusion cysts are benign cutaneous lesions caused by dermal or subdermal implantation and proliferation of epidermal squamous epithelium as a result of trauma or surgery. They are typically located on the scalp, face, trunk, neck, or back; however they can be found anywhere on the body. Lesions are asymptomatic unless complicated by rupture, malignant transformation to squamous cell carcinoma, or infection at which point they can clinically appear as more sinister pathologies. We present the case of a 45-year-old laborer with a ruptured epidermal inclusion cyst, manifesting clinically and radiographically as a malignancy. Following MRI, definitive surgical management may appear to be a logical progression in management of the patient. This case however is a good example of why meticulously following surgical protocol when evaluating an unknown soft tissue mass is imperative. By following protocol, an alternate diagnosis was made and the patient has since gone on to a make a full recovery without life transforming surgery.

## 1. Introduction

A 45-year-old male presented to the orthopaedic outpatient department with an 18-month history of a gradually growing mass on the middle finger of his right dominant hand. The mass had grown at an increased rate over the previous 6 months culminating in self-referral to the emergency department after acute pain affecting his ability to complete work as a laborer at the local port. The patient identified a crush injury to his fingers involving a fridge approximately 6 months earlier; concluding the mass had extended in size from this time. After a failed attempt at aspiration the patient was discharged on oral antibiotics with orthopaedic follow-up. He reports no significant past medical history; however he smokes 10–15 cigarettes per day.

On examination, a fusiform swelling of his right middle finger was present centred on a tender mass on the radio-volar aspect of the middle phalanx. There was no evidence of infection or vascular disturbance; however paraesthesia was noted distal to the mass on the ulnar aspect of his finger. Flexion at the interphalangeal and metacarpophalangeal joints was restricted secondary to pain and mass effect of the lesion.

**Figure 1:**
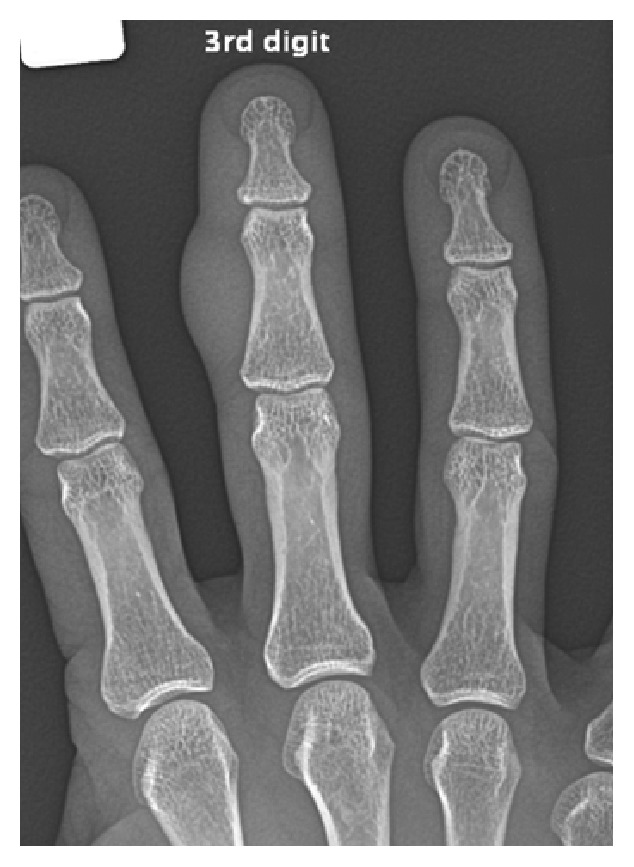
AP radiograph showing large fusiform soft tissue swelling of right middle finger at a level of the middle phalanx.

X-ray demonstrated a radial soft tissue swelling without bony involvement (Figure 1) whilst an ultrasound scan demonstrated marked subcutaneous oedema and thickening of the flexor tendon with synovial thickening of the PIPJ. No drainable focal fluid collection or foreign body was demonstrated.

**Figure 2:**
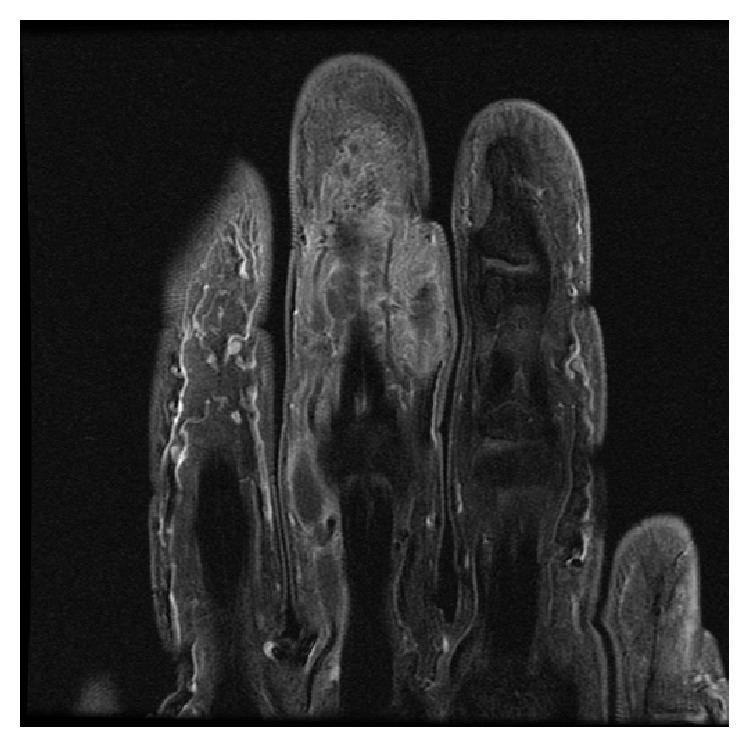
Proton density fat saturated coronal image showing a poorly defined lesion extending to the web space.

**Figure 3:**
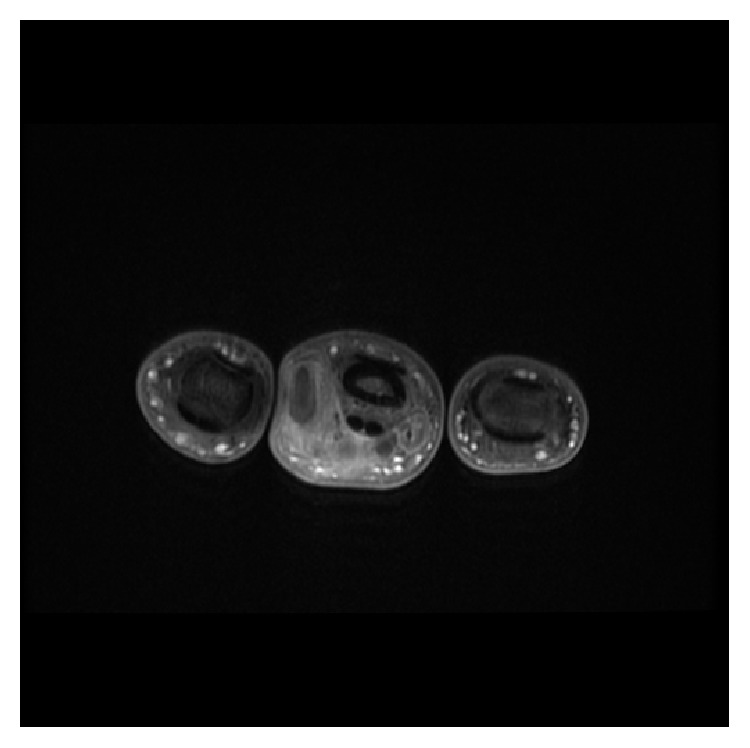
Axial proton density fat saturated sequence showing a mass extending hemicircumferentially around the flexor tendon of the middle phalanx.

**Figure 4:**
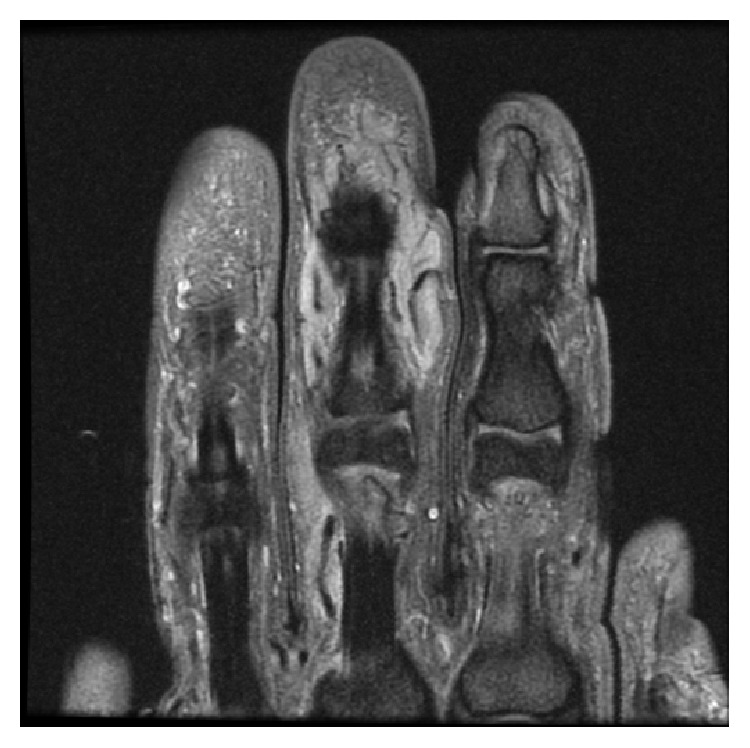
Coronal T1 fat saturated image after contrast showing central area of nonenhancement (necrosis/cystic content) and web space extension.

**Figure 5:**
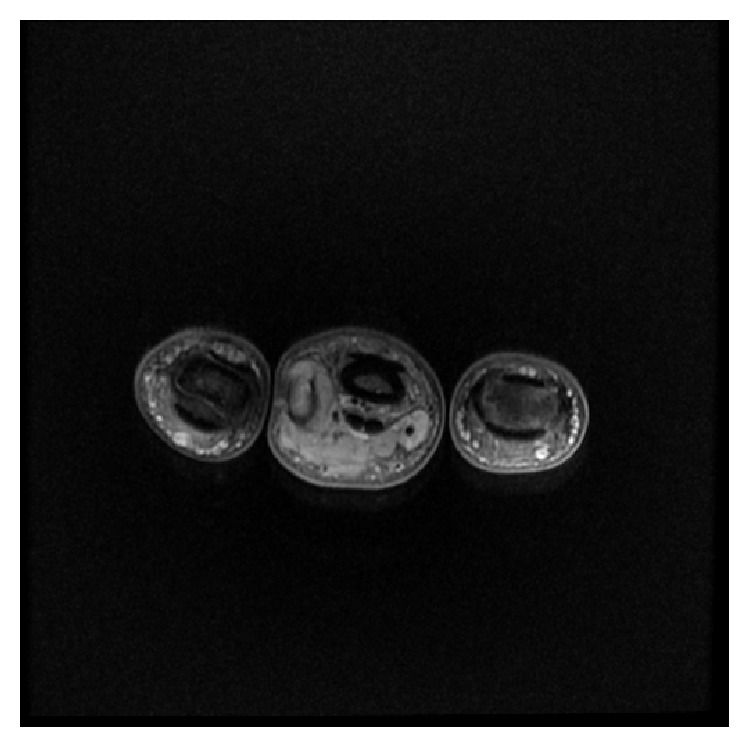
Axial T1 fat saturated after contrast.

**Figure 6:**
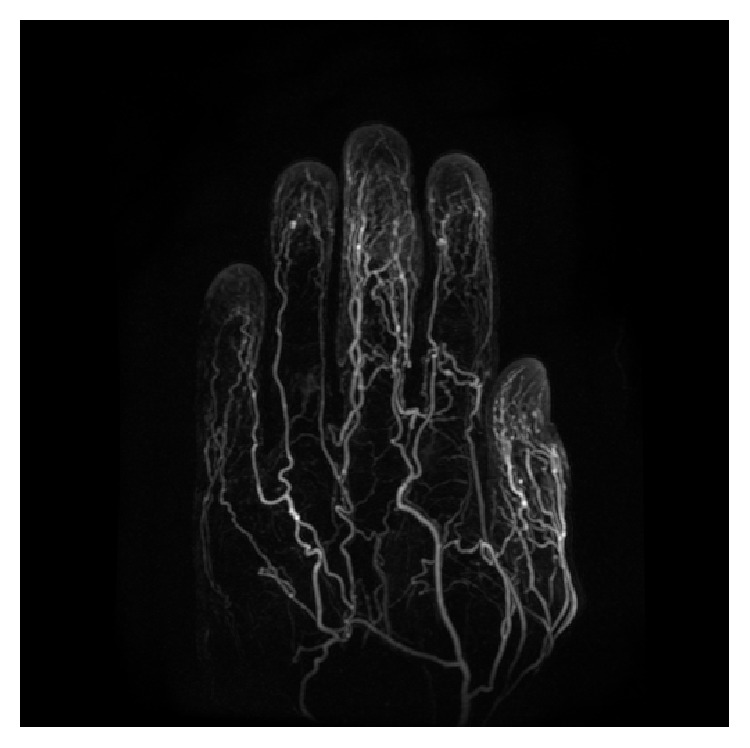
3D postcontrast Time Resolved Imaging of Contrast Kinetics (TRICKS) angiogram showing vascularity of the lesion.

Urgent magnetic resonance imaging (MRI) with contrast was requested demonstrating an extensive poorly defined infiltrating soft tissue mass around the middle phalanx, of intermediate T1 signal (Figures 2 and 3) and high T2 signal (Figures 4 and 5). The large hemicircumferential component abutting the flexor tendon is noted whilst a central tongue extends distally. A lobulated proximal extension is also noted extending just short of the 2nd web space. There was moderate enhancement with significant areas of central nonenhancement, most in keeping with malignancy. The differential diagnosis includes synovial sarcoma or epithelioid sarcoma. There was increased vascularity to the lesion (Figure 6). There was no bone or joint involvement.

The patient proceeded with incisional biopsy prior to likely ray amputation. A mid-lateral radial incision was made over the middle phalanx and four large pieces of tan coloured, friable, abnormal tissue were resected and sent for histology. Wound swabs and a small amount of necrotic tissue was sent for microscopy, culture, and sensitivity. Staging computed tomography (CT) of chest, abdomen, and pelvis showed no evidence of metastases. Microbiology samples identified light growths of* Staphylococcus warneri*,* Staphylococcus capitis*, and* Staphylococcus epidermidis* susceptible to flucloxacillin.

Pathology reports showed sections of fibrovascular connective tissue with a small area of associated hyperkeratotic stratified squamous epithelium. Fragments of calcified debris were visible within an extensive foreign body type granulomatous inflammatory cell infiltrate. Findings were in keeping with a ruptured epidermal inclusion cyst with secondary inflammatory response.

**Figure 7:**
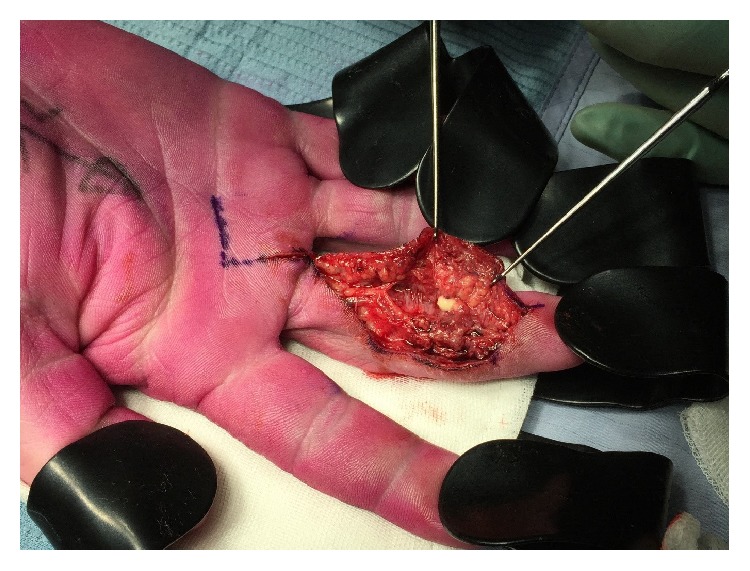
A central area of white pus can be seen whilst the necrotic tissue of the mass on the radial aspect of the digit extends into the web space.

An excision of the soft tissue mass was performed after confirming benign nature of the mass with frozen section analysis. Intraoperatively a 3 mm sharp foreign organic body was identified in the mass, around an area of pus and necrosed tissue (Figure 7).

The patient has since proceeded to make a full recovery and has returned to full time work.

## 2. Discussion

Epidermal inclusion cysts are subcutaneous lesions caused by dermal or subdermal implantation and proliferation of epidermal squamous epithelium as a result of trauma or surgery. They are typically located on the digits, scalp, face, trunk, neck, or back; however they can be found anywhere on the body. Occlusion of pilosebaceous units, human pappilomavirus 57, and HPV 60 infection are rare but significant alternate pathogenesis. Most patients present with an asymptomatic or incidental mass unless complicated by rupture, malignant transformation to squamous cell carcinoma, or infection [1, 2].

Sonographically, the cysts usually appear as well as circumscribed hypoechoic masses. MRI scanning is the investigation of choice. T1 weighting shows low or intermediate signal whilst T2 weighting shows high signal. Differential diagnosis should include neurogenic tumours, Myxoid tumours, dermatofibrosarcomas, nodular fasciitis, and ganglion cysts [2].

As with any lesion, it is imperative to meticulously follow surgical protocol in the diagnosis and management stages to minimise adverse outcomes, incomplete resection, seeding, bleeding, and infection. Most errors in management of an unknown lesion occur from incomplete or inappropriate presurgical diagnosis [3].

Initial assessment should begin with comprehensive history and physical examination. Social history of environmental exposures and smoking status can be key whilst systemic features such as fever, weight loss, and malaise are infrequent but unforgiveable if missed.

Large rapidly growing lesions should invoke immediate concern whilst tenderness is often pathognomonic of infection and inflammation or less commonly malignant infiltration. Lesions that are superficial, cystic, or less than 5 cm in size are likely to be benign whilst deep lesions larger than 5 cm have a higher malignant potential. Lymph node examination is imperative [3].

Biplanar radiographs and ultrasound are suitable and cost effective initial investigations; however, magnetic resonance imaging is the imaging investigation of choice should any concerning features be raised. Biopsy and pathological evaluation should remain the last events in the evaluation of a soft tissue mass. It is good practice to discuss the biopsy procedure with pathology and radiology specialists prior to procedure to avoid tumour seeding and consideration of limb salvage procedures. Biopsy whether open or closed should be performed adhering to the following principles [3]:

1. Careful consideration of approach to avoid further neurovascular or compartmental contamination.
2. Lesions extending to bone which should be sampled from soft tissue to avoid increasing the risk of pathological fractures.
3. The track which should be excisable en bloc with the tumour.
4. Avoidance of haematoma collection.

CT staging of malignant lesions should be undertaken with chest, abdomen, and pelvic imaging to investigate metastases and is useful as an adjuvant to MRI for delineating tumour matrix and cortical destruction [3].

## 3. Conclusion

It is likely that the small thorn-like structure identified on removal of the mass was responsible for an unnoticed penetrating injury to the finger some time earlier, implanting dermis deep in the digit. The incident with the fridge may have resulted in rupture of the ensuing epidermal inclusion cyst, propagating its extension along the length of the finger. This rupture in combination with chronic low grade infection secondary to foreign body and constant aggravation due to the physical nature of the patient's job resulted in the semiacute deterioration in symptoms and presentation.

Complication of epidermal inclusion cysts with rupture, infection, or malignant transformation compounds clinical diagnosis. This case is a good example of why meticulously following surgical protocol when evaluating a soft tissue mass is imperative. Following MRI, definitive surgical management may appear to be a logical progression in management of the patient. In this case, a clinically and radiographic diagnosis of synovial sarcoma would have resulted in ray amputation extending into the hand. By following protocol, an alternate diagnosis was made and the patient has since made a full recovery without life transforming surgery.
